# Meeting report of the 2017 KidGen Renal Genetics Symposium

**DOI:** 10.1186/s40246-018-0137-7

**Published:** 2018-01-30

**Authors:** Kushani Jayasinghe, Cathy Quinlan, Zornitza Stark, Chirag Patel, Matthew G. Sampson, Moin Saleem, Andrew J. Mallett, on behalf of the KidGen Collaborative

**Affiliations:** 1 Department of Nephrology, Monash Medical Centre, Melbourne, Australia; 2 KidGen Renal Genetics Flagship, Australian Genomic Health Alliance, Melbourne, Australia; 3 Murdoch Children’s Research Institute, Melbourne, Australia; 4 Department of Paediatric Nephrology, Royal Children’s Hospital, Melbourne, Australia; 5 Department of Pediatrics, University of Melbourne, Melbourne, Australia; 6 Genetic Health Queensland, Royal Brisbane and Women’s Hospital, Brisbane, Australia; 7 Department Of Pediatrics-Nephrology, University of Michigan School of Medicine, Ann Arbor, USA; 8 Bristol Renal, Bristol Medical School, University of Bristol, Bristol, UK; 9 Faculty of Medicine, The University of Queensland, Brisbane, Australia; 10 Kidney Health Service and Conjoint Renal Research Laboratory, Royal Brisbane and Women’s Hospital, Butterfield Street, Herston, Brisbane, Queensland 4029 Australia

**Keywords:** Inherited kidney disease, Nephrogenetics, Renal genetics

## Abstract

The 2017 KidGen Renal Genetics Symposium was held at the Royal Children’s Hospital and Murdoch Children’s Research Institute, Melbourne, from 6 to 8 December 2017. This meeting addressed clinical, diagnostic, and research aspects of inherited kidney disease. More than 100 clinicians, researchers, and patient representatives attended the conference. The overall goal was to improve the understanding and direction of genomics in renal medicine in Australia and discuss barriers to the use of genomic testing within this area. It also aimed to strengthen collaborations between local, state, and global research and diagnostic and clinical groups.

## Introduction and background to the meeting

There has been a rapid change and progress in the field of inherited kidney disease and nephrogenetics in recent years, and this has across clinical, diagnostic, and research domains. Within an Australian and Australasian perspective, there have been not only challenges but also opportunities related to this translational process. The obstacles of geography and distance are countered by nationally and regionally coordinated genomic efforts such as Australian Genomics, Melbourne Genomics, and the Queensland Genomic Health Alliance. The annual KidGen Renal Genetics Symposium provides an opportunity to bring together a diversity of clinicians, researchers, and patient representatives from around Australia, Australasia, and the global community. This enables a unique opportunity to better understand the renal genetics implementation landscape while updating on the latest progress within a strong nexus of collaboration focused on this particular emerging subspecialty field.

## The renal genetics multidisciplinary workshop

This interactive half-day clinical workshop, convened by Dr. Chirag Patel from Genetic Health Queensland, was designed primarily for nephrologists, geneticists, and genetic counselors new to the clinical practice of renal genetics. Thirty participants attended the workshop, with small groups of six including a facilitator. Participants in each group comprised of a mixture of professionals, which included adult and pediatric nephrologists, genetic counselors, and clinical geneticists, reflecting the typical composition of a multidisciplinary clinic or meeting. Case-based scenarios were discussed including pertinent issues in each case, differential diagnosis, information required, counseling and ethical issues, and genetic testing indications and results. All participants also took part in a survey to evaluate their initial understanding of genomics and confidence in renal genetics cases, and these survey findings will be analyzed as part of an implementation science analysis of renal genetics in Australia.

## Providing a map of services available to date

**Fig. 1:**
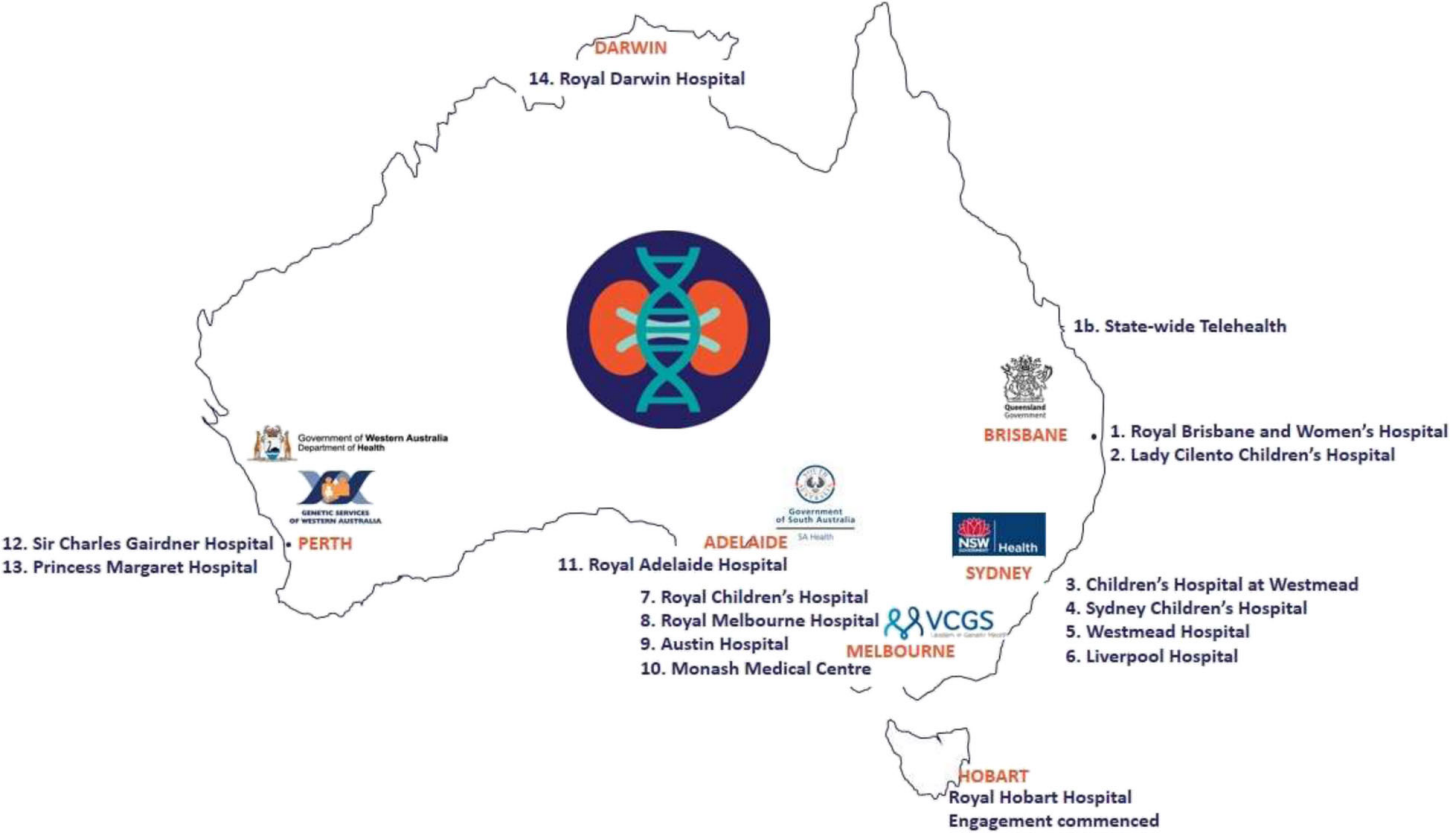
Location of KidGen-affiliated Renal Genetics Clinics as of 7 December 2017

Dr. Andrew Mallett, the National Director of the KidGen Collaborative and the Australian Genomics Health Alliance (AGHA) Renal Genetics Flagship, presented on the renal genetics services and research opportunities in Australia. The KidGen Collaborative continues to grow and evolve. In the last year, services have commenced in South Australia and Western Australia (Fig 1). Multidisciplinary renal genetics clinics will soon be underway in Darwin and Tasmania, with the aim to provide access to 90% of the Australian population over the next 12 months. Dr. Mallett also described the challenges of recruiting to the Australian Genomics renal flagship. Potential participant recruitment rates have not been as high as projected, and research looking into reasons for this is being conducted via an implementation science project. Another exciting new venture is the Australian Genomics “wHole genome Investigation to iDentify unDEtected Nephropathies (HIDDEN) Flagship” which has recently secured funding to identify undetected genetic nephropathies using whole genome sequencing. Currently, 5% of patients with end-stage kidney disease (ESKD) have an unknown cause of their renal failure. With the cost of genome sequencing decreasing, there is an opportunity to remediate this. The immediate aim is to evaluate 200 participants with unexplained ESKD over the next 24 months. He also highlighted that significant prospective studies evaluating the cost-effectiveness of genomic testing are occurring in Australian cohorts, which will be crucial in securing sustainable funding for genomic testing in routine healthcare.

## Rare inherited kidney disease registries

This session focused on the opportunities and challenges of developing rare disease registries. Professor Moin Saleem, Head of Bristol Renal, a world renowned glomerular disease research group, presented on the United Kingdom (UK) approach to rare inherited kidney disease. Rare inherited kidney disease refers to diseases that have a prevalence of 1 in 2000 [1]. Up to 10% of the population will have a rare disease, and 80% of rare diseases will have a genetic basis. The UK renal community has developed perhaps the world leading system for ongoing collation of cohorts of patients with renal rare disease, called renal RaDaR (www.rarerenal.org). This is a web-based and nationally inclusive program, which allows information to build in a bottom-up fashion, driven by interested and enthusiastic stakeholders, professionals, and patients. This registry was developed utilizing the information technology (IT) infrastructure of the UK renal registry. The original target of the registry was to recruit 500 patients, whereas currently, there are over 15,000 patients comprising 25 patient groups and 85 renal units.

Professor Saleem described the importance of understanding rare disease using modern biological advances. In order to stratify and treat patients appropriately, we must move from descriptive classifications to molecular levels of understanding. To do this, it is crucial to recruit as many patients as possible in order to facilitate studies with sufficient analytical power. The RaDaR registry has also provided the opportunity to create rare disease groups and facilitated the development of evidence-based care pathways and further empower patients. There are tremendous opportunities that arise from such registries, and other countries can benefit from the lessons learned from the UK renal registry.

## Genomic profiling and molecular insights in nephrotic syndrome

Keynote speakers presented on the benefits of deep clinical and genomic profiling in nephrotic syndrome. Professor Saleem discussed the importance of discovering robust biological biomarkers that identify patients with circulating factor disease. For example, there is strong evidence for a role of a circulating factor in the pathogenesis of idiopathic nephrotic syndrome (iNS) [2, 3]. Previous work has suggested a role for protease-activated receptor (PAR-1) [4]. His group used a model whereby a transgenic mouse was developed that expressed a podocyte-specific constitutively active form of PAR-1 in order to replicate overexposure to a circulating protease. The developmental PAR-1 active mice were born normal and died consistently between the ages of 39 and 45 days. Proteinuria developed from 14 days and markedly increased over time. Electron microscopy (EM) analysis showed a significantly thickened glomerular basement membrane (GBM) and foot process effacement. This demonstrated a clear role for the PAR-1 receptor in proteinuria and strengthened the hypothesis that circulating factor(s) may act via this receptor. Additionally, this is a novel model for circulating factor nephrotic syndrome (NS) to test therapies in vivo. Professor Saleem presented this work at the recent American Society of Nephrology Kidney Week in November 2017.

Dr. Matthew Sampson, a pediatric nephrologist and researcher at the University of Michigan, spoke on the use of population genetics to interrogate monogenic nephrotic syndrome diagnosis. His laboratory combines innovative genomic technologies, statistical genetics, computational genetics, and bioinformatics to elucidate the role of genomic variation in renal disease. Genomic discovery in nephrotic syndrome has allowed insight into biology of disease, clinical correlates such as prognosis and treatments, and prevalence estimates of disease.

Monogenic nephrotic syndrome is a serious form of NS, and these patients often have a continued decline in kidney function that is resistant to immunosuppressive therapy. [5–9]. There are over 60 causative genetic mutations of steroid resistant nephrotic syndrome (SRNS) [10]. Numerous publications have reported robust genotype-phenotype correlations in monogenic NS and their substantial prevalence. Previously, diagnostic genetic screening involved sequencing one or two genes most likely to harbor causal variants in patients with a high pretest probability of having a genetic form of nephrotic syndrome. Once found, other evidence was collected to support causality. Nowadays, genomic technologies are rapidly evolving, enabling sequencing to become much more readily available. Hence, there is a widespread enthusiasm from all stakeholders to expand sequencing efforts in affected patients to make a diagnosis of “monogenic NS.” However, there is a need to assess the generalizability of prevalence estimates and clinical correlates previously reported from selected cohorts now that more patients and more genes can be studied. One aspect of this is the recognition from large-scale sequencing projects in reference populations that the prevalence of putatively fully penetrant mutations in the general population can be orders of magnitude higher than the reported disease prevalence***.***

The NEPTUNE study [11] is a North American cohort of NS patients that provided a laboratory to gain insights on the challenges of making a monogenic diagnosis in previously understudied populations. Patients with NS were enrolled at the time of the first biopsy and independent of age, ancestry, and response to immunosuppression. They were then followed longitudinally. In a paper by Sampson et al. [12], 312 patients underwent targeted sequencing of 21 genes implicated in SRNS. These were compared to the 1000 Genomes Project (1000G) reference population. A typical pathogenicity filter identified causal variants for NS in 4.2% of patients and 5.8% of subjects from the 1000G. The group then devised a more stringent pathogenicity filtering strategy, implicating monogenic NS in 2.9% of NEPTUNE patients and reducing background prevalence of causal variants to 1.5% in the 1000G. Interestingly, patients with putative monogenic NS in NEPTUNE achieved complete remission of proteinuria at similar rates to those without a monogenic diagnosis.

The 1.5% background prevalence suggests that some variants, even if found in patients, might be either incorrectly implicated as pathogenic. In addition, there may be a greater degree of incomplete penetrance of these variants than previously appreciated. Therefore, in an unselected cohort, it may be accurate to consider a monogenic diagnosis from a probabilistic and not a deterministic perspective.

## Functional genomics

Professor Melissa Little, the theme Director of Cell Biology at Murdoch Children’s Research Institute (MCRI) in Melbourne and program leader of Stem Cells Australia, discussed the clinical and research applications for induced pluripotent stem cells and their application in kidney disease. Advances in next-generation sequencing have improved diagnosis rates for families with inherited kidney disease; however, a causative gene is identified in only 40% of cases [13]. While novel potential causative gene associations are being identified, these still require functional validation**.** The use of induced pluripotent stem cells (iPSC) is one approach that has the potential to determine the functional significance of previously un-described gene variants. Professor Little and her team developed a protocol for the successful differentiation of human pluripotent stem cells to a kidney organoid [14, 15]. This results in the formation of a complex organoid containing all cellular components anticipated for a first trimester human kidney. Professor Little also presented data illustrating how we can replicate the disease phenotype of an inherited kidney disease. By modeling heritable kidney disease and screening of toxicity and efficacy, we are now able to move towards applying stem cells to cellular therapy. Renal replacement therapy through iPSC-derived cells is still some time away, mainly due to barriers of scale and vascular supply.

## Challenges in genomic medicine

This session focused on current challenges in implementing genomic medicine into clinical practice as well as the innovative opportunities and solutions achieved to date. Elly Lynch, a clinical project manager at Melbourne Genomics Health Alliance (MGHA), highlighted the potential health implications from incidental or secondary results that arise from genomic sequencing. This refers to variants found incidentally through genomic sequencing that are not related to the aim of the test but may still have potential health implications and clinical significance [16].

In 2013, the American College of Medical Genetics and Genomics (ACMG) published a policy statement that emphasized the importance of reporting of incidental results [17]. In 2014, the ACMG revised these recommendations to include options for individuals to opt out of being informed of incidental findings. The ACMG Board of Directors created the ACMG Secondary Findings Maintenance Working Group (SFWG) in 2014 to define and implement a process for updating a minimum list of secondary findings that would be updated and refined regularly. The 2016 update statement concluded that “Informed consent is necessary, and reporting of secondary findings should be optional” [18]. However, surprisingly, the research into this area has reflected that often, patients want to be informed of secondary findings, even when limited treatment options are available [19].

Professor Kathryn North, co-chair of the Global Alliance for Genomics and Health (GA4GH) and lead of the Australian Genomics Health Alliance, highlighted the challenges of the implementation of genetic services in Australia. She provided insight into the complex and fragmented structure of the Australian healthcare system. Federal funding for genetic services is currently limited to about 30 tests and genomic testing being supported by clinical services at a state level. This highlighted the importance of linking and collaborating state-based expertise and investment with the formation of national flagships in order to facilitate research, map disparity, and inequity and allow coordinated collaboration between states.

She also highlighted the importance of global collaboration. In October 2017, Australian Genomics was nominated a Driver Project of the GA4GH, an international, nonprofit alliance of over 470 organizations across over 45 countries. Australian Genomics will serve alongside Genomics England and the *All of Us* research program to enable GA4GH to deliver its new 5-year strategic plan to enable the responsible sharing of clinical-grade genomic data by 2022.

## The benefits of rapid genomic testing in acute pediatrics

A/Prof Zornitza Stark, clinical geneticist at the Victorian Clinical Genetics Service (VCGS) and clinical research fellow with Australian Genomics, demonstrated the significant clinical and cost benefits from rapid whole exome sequencing (rWES) in a prospective cohort of 40 acutely unwell pediatric patients recruited through the Melbourne Genomics Health Alliance in 2016–2017. In the course of the project, the overall time-to-result decreased tenfold from 109 to 9 days (median 16 days). This required whole-of-system change, with over 20 changes implemented to clinical and laboratory processes to accelerate result delivery. Of the 40 patients who underwent rWES, 52% received a genetic diagnosis, and 57% of those diagnosed had a change in management as a result, including the provision of life-saving treatment, avoidance of invasive biopsies, and redirection towards palliative care. Rapid exome sequencing was also very cost-effective, with cost savings from improved management conservatively estimated to be around AU$550,000 (Stark Z, Lunke S et al: Meeting the challenges of implementing rapid genomic testing in acute pediatric care, submitted).

## Genomics in specific ethnic groups

This session focused on genetic variation in patients with chronic kidney disease within Indigenous populations, as well as the lessons learned and challenges of research in various ethnic groups. Matthew Sampson presented on the racial disparity of ESKD in the American population. African-Americans make up only 13% of the US population yet make up 32% of all Americans with ESKD [20]. African-Americans who carry two variant alleles within apolipoprotein L1 (*APOL1*) are classified as having a high-risk (HR) genotype [21]. People with HR genotype have an increased risk of focal segmental glomerulosclerosis and chronic kidney disease compared with those with a low-risk (LR) genotype. Children from two cohorts with HR genotype were characterized (Chronic Kidney Disease in Children (CKiD) and NEPTUNE), which found that children with a HR genotype had a more aggressive form of glomerular disease [22].

William Wong, a pediatric nephrologist at Starship Children’s Hospital, Auckland, discussed the congenital nephrotic syndrome in Maori children. Maori children tend to have a milder course compared to infants with Caucasian background. *Nephrin* mutation analysis in 19 surviving CNS patients revealed a variety of different mutations in *NPS1*, with Caucasian infants demonstrating completely different mutations to Maori infants [23]. 

Madhivanan Sundaram, a nephrologist at Royal Darwin hospital, presented on the practical challenges in the diagnosis and management of inherited kidney disorders in remote Indigenous patients. Due to language barriers, cultural barriers, and scarcity of resources in remote communities, there are many challenges nephrologists face in this population group. Some of these include difficulties in pedigree analysis, obtaining informed consent, and reluctance to participate in research. Some solutions to overcoming these obstacles include involving genetic counselors from indigenous groups, increased use of interpreters, and close links with local community groups and clinics.

## Panel discussion: “You can’t always get what you want – whole genome sequencing, whole exome sequencing and renal gene panels”

Panelists:

A/Prof Julie McGaughran: Director of Genetic Health Queensland, Royal Brisbane and Women’s Hospital, Brisbane

Dr. Cas Simons: Adjunct Senior Fellow, Institute for Molecular Bioscience, University of Queensland, Brisbane

Dr. Amali Mallawaarachchi: Adult Nephrologist and Clinical Genetics Fellow, Liverpool Hospital, Sydney

A/Prof Bruce Bennetts: Associate Professor and Head of Department of Genetic Medicine, Children’s Hospital at Westmead, Sydney

Dr. Sebastian Luke: Head of the Translational Genomics Unit, Victorian Clinical Genetics Services and Murdoch Children’s Research Institute, Melbourne

A/Prof Zornitza Stark: Clinical Geneticist, Victorian Clinical Genetics Services and Australian Genomics Health Alliance, Melbourne

The panel discussion allowed a passionate group of physicians, geneticists, scientists, and counselors the opportunity to ask thought-provoking questions and participate in open-minded discussions on the major topics surrounding genomics in renal medicine. Urgent unmet needs and challenges in the future were addressed. Issues raised included the “best test” in the eyes of laboratory and research scientists versus clinical geneticists and renal physicians and the balance between cost of sequencing and selecting the optimal test for the patient. Overall, the consensus among panel members was that the current cost of whole genome sequencing is prohibitive to its clinical use and hence does not justify the cost of doing this over whole exome sequencing or targeted panel. Furthermore, we do not know enough about the whole genome yet to be able to fully interpret the sequencing results. It is still unclear who should be the gatekeepers to genetic testing in the future. Should any doctor be able to order genetic tests? Who is responsible for counseling patients? Currently, this differs from state to state, but in general, patients seen in renal genetics clinics are discussed at multidisciplinary meetings to ensure appropriate test ordering. The general consensus was that genetic testing should primarily be ordered if it would change patient management.

## Conclusions and outcomes

The KidGen Renal Genetics Symposium is currently one of the few dedicated renal genetics meetings to be held internationally on an annual basis. In 2017, the 5th annual meeting brought together scientific researchers, clinical researchers, adult and pediatric nephrologists, patient representatives, clinical geneticists, and genetic counselors. This model exemplifies how research collaboration should be facilitated, as it brings together the key members and stakeholders that can advance the field of renal genomics. This is of utmost importance to benefit patients and families with inherited kidney disease. The symposium provided not only the increased awareness of the challenges of genomics we face now and into the future but also the promise of insights and advances that are continuously being gained.

## Abbreviations

1000G: 1000 Genomes Project
ACMG: American College of Medical Genetics and Genomics
AGHA: Australian Genomics Health Alliance
ESKD: End-stage kidney disease
EM: Electron microscopy
GA4GH: Global Alliance for Genomics and Health
GBM: Glomerular basement membrane
HIDDEN: wHole genome Investigation to iDentify unDEtected Nephropathies
HR: High risk
iPSC: Induced pluripotent stem cells
IT: Information technology
iNS: Idiopathic nephrotic syndrome
LR: Low risk
MCRI: Murdoch Children’s Research Institute
MGHA: Melbourne Genomics Health Alliance
NS: Nephrotic syndrome
rWES: Rapid whole exome sequencing
SFWG: Secondary Findings Working Group
SRNS: Steroid resistant nephrotic syndrome
UK: United Kingdom
VCGS: Victorian Clinical Genetics Service
